# Linear dose-response relationship between TyG-WC index and diastolic dysfunction: insights from restricted cubic spline and machine learning analysis

**DOI:** 10.3389/fendo.2026.1870221

**Published:** 2026-07-20

**Authors:** Yanbo Xia, Jin Wang, Zhiwei Huang, Jing Bai, Junxiang Liu, Jirui Cai, Bing He, Li Guo, Qiaotao Xie, Haoran Wang

**Affiliations:** Luohe Central Hospital, Luohe Medical College, Luohe, China

**Keywords:** central adiposity, diastolic dysfunction, insulin resistance, machine learning, TyG-WC

## Abstract

**Background:**

While diastolic dysfunction (DD) is a critical heart failure precursor linked to metabolic stress, the specific association between the promising triglyceride-glucose waist circumference (TyG-WC) index and DD remains insufficiently characterized under contemporary diagnostic standards. This study aimed to evaluate the association between the TyG-WC index and the risk of DD in a community-based population of middle-aged and older adults.

**Methods:**

In this cross-sectional study of 1,413 participants, DD was adjudicated according to the age-stratified echocardiographic criteria. Multivariable logistic regression and restricted cubic splines (RCS) were employed to assess independent associations and dose-response relationships. Furthermore, machine learning techniques, including LASSO regression for feature selection and Random Forest models interpreted by Shapley Additive exPlanations (SHAP), were utilized to quantify the predictive contribution of TyG-WC.

**Results:**

After full adjustment for demographics, blood pressure, and clinical comorbidities, TyG-WC was independently associated with an increased risk of DD (OR = 1.002, 95% CI: 1.001-1.004, *P* < 0.001). Individuals in the high TyG-WC group (**≥**776.5) faced a significantly higher risk of DD compared to the low group (OR = 1.656, 95% CI: 1.266-2.166, *P* < 0.001). RCS analysis confirmed a significant linear dose-response relationship (*P*_overall_<0.001), indicating that the probability of DD escalates with increasing TyG-WC. The full predictive model achieved an AUC of 0.687, demonstrating incremental value beyond traditional risk factors. Subgroup analyses demonstrated the robustness of these findings, with significant interactions observed for hypertension and diabetes status. Machine learning identified TyG-WC as an independent contributor for DD, with SHAP values consistently linking higher index levels to increased risk.

**Conclusions:**

Elevated TyG-WC is independently associated with an increased risk of DD. As a cost-effective and easily accessible marker of cardiometabolic stress, TyG-WC may serve as a practical screening tool for the early identification and risk stratification of subclinical heart failure.

## Introduction

Cardiovascular diseases (CVDs) constitute the leading global burden of disease, with heart failure (HF) representing a primary and increasingly prevalent challenge within this clinical spectrum (1, 2). Diastolic dysfunction (DD), which involves impaired ventricular relaxation and decreased myocardial compliance, is now recognized as a fundamental pathophysiological precursor to heart failure with preserved ejection fraction (HFpEF) (3, 4). The recent 2025 American Society of Echocardiography (ASE) update has significantly refined the diagnostic landscape for DD by introducing age-stratified thresholds for early diastolic mitral annular velocity (e’) (5) This evolution in clinical guidelines underscores the necessity of employing age-adjusted metrics to detect subclinical myocardial impairment early, potentially forestalling the progression to symptomatic heart failure (6).

Insulin resistance (IR) has emerged as a central driver in the development of metabolic and cardiovascular disorders, contributing to adverse structural and functional remodeling of the heart (7, 8). While the hyperinsulinemic-euglycemic clamp remains the diagnostic gold standard for IR, its clinical utility is limited by high costs and procedural complexity (9). Consequently, the triglyceride-glucose (TyG) index has gained prominence as a reliable and accessible surrogate for IR (10). To enhance its predictive accuracy, recent research has integrated anthropometric measures, leading to the development of the triglyceride (TG), fasting plasma glucose (FPG), waist circumference (WC) index (11). By combining metabolic markers with central adiposity data, triglyceride-glucose waist circumference (TyG-WC) offers a more holistic assessment of “metabolic-mechanical stress” on the myocardium than either lipid profiles or waist circumference alone (12).

Despite growing evidence linking IR to cardiac functional decline, the specific association between the TyG-WC index and diastolic function remains insufficiently characterized (13, 14). Previous studies have predominantly utilized traditional obesity markers like Body Mass Index (BMI) or individual lipid fractions, which may fail to capture the synergistic impact of insulin dysregulation and visceral fat on myocardial relaxation (15, 16). Furthermore, there is a distinct lack of large-scale, community-based investigations evaluating TyG-WC in the context of the newly implemented 2025 ASE age-stratified diagnostic framework.

Consequently, the objective of the cross-sectional study was to examine the association between TyG-WC and the risk of DD within a middle-aged and elderly population, utilizing data from the Luohe City Longitudinal Investigation of Osteoarthritis and Cardiovascular Health Status. We hypothesized that an elevated TyG-WC index would be independently predictive of a higher prevalence of DD and poorer echocardiographic performance. By validating TyG-WC as a potential clinical marker, this research aims to provide a practical and cost-effective strategy for early cardiovascular risk stratification and the prevention of HFpEF.

## Methods

### Study population

This cross-sectional analysis was conducted utilizing baseline data from the Longitudinal Investigation of Osteoarthritis and Cardiovascular Health Status cohort, a prospective observational cohort (17). The parent study was carried out in Luohe, China, and its surrounding areas between November 2023 and November 2024, primarily investigating the relationship between osteoarthritis and major adverse cardiovascular events. The study protocol was approved by the Institutional Review Board of Luohe Central Hospital (No. 2023010) and registered prospectively with the Chinese Clinical Trial Registry (ChiCTR2300071492). The current study adhered to the Strengthening the Reporting of Observational Studies in Epidemiology (STROBE) guidelines (18).

From the initial cohort, we screened participants who had completed both comprehensive laboratory biochemical profiling and transthoracic echocardiography. Exclusion criteria were defined as (1): age under 18 years (2); insufficient serum lipid or anthropometric data for calculating the TyG-WC index (missing TG or WC) (3); incomplete echocardiographic metrics for diastolic function grading; and (4) pregnancy or the presence of terminal illnesses. Participants with missing data on any of the key variables were excluded from the primary analyses. Following these exclusions, a total of 1,413 participants were eligible for the final analytic sample.

### Measurements

Serum total cholesterol (TC) was detected using the Cholesterol Oxidase-Peroxidase method, triglyceride (TG) was measured via the Glycerol-3-Phosphate Oxidase-Peroxidase enzymatic colorimetric method, low-density lipoprotein cholesterol (LDL-C) was determined by a direct surfactant clearance method, high-density lipoprotein cholesterol (HDL-C) was tested with a direct selective inhibition method, and glucose was quantified by the hexokinase method; all lipid assays were performed with commercial kits purchased from Leadman Biochemistry Co., Ltd. (Beijing, China), while the fasting plasma glucose (FPG) assay kit was supplied by Maccura Biotechnology Co., Ltd. (Chengdu, China), and all biochemical indicators were analyzed on a Beckman AU-5831 automatic biochemical analyzer. The primary exposure, TyG-WC, was utilized as a composite metabolic marker of lipid-related stress. TG and FPG values were converted from mmol/L to mg/dL before calculating the TyG index. The exact equations added to the Methods section are as follows: TG (mg/dL) = TG (mmol/L) × 88.57; FPG (mg/dL) = FPG (mmol/L) × 18.02; TyG was calculated as ln[TG (mg/dL) x FPG (mg/dL)/2], and TyG-WC was calculated by integrating the TyG with WC according to the formula: TyG-WC = TyG × WC (cm) (19). To ensure statistical robustness and evaluate potential dose-response effects, TyG-WC was analyzed as both a continuous variable and a categorical variable divided into quartiles (Q1–Q4), with the lowest quartile (Q1) serving as the reference. The primary outcome was DD, which was adjudicated based on the 2025 ASE updated guidelines (5). All transthoracic echocardiographic examinations were performed by trained sonographers using a Philips EPIQ 7 system (Philips Healthcare, Amsterdam, Netherlands) equipped with an S5–1 phased-array transducer (frequency range: 1–5 MHz).

Tissue Doppler imaging (TDI): Mitral annular e′ velocity was acquired in the apical four-chamber view using the TDI preset (optimized for low-velocity, high-amplitude signals). A pulsed-wave sample volume of 5–10 mm was placed at both the septal and lateral insertion sites of the mitral leaflets, with the angle of interrogation aligned as parallel as possible to annular motion. The velocity scale and zero baseline were adjusted to display the full spectral signal, and a sweep speed of 100 mm/s was used to obtain sharp spectral waveforms without spikes or feathering. The average e′ velocity was calculated as the mean of septal e′ and lateral e′ measurements, consistent with current guideline recommendations. Intra-observer and inter-observer reproducibility of average e′ were assessed in a randomly selected subset of 50 participants. Intra-observer intraclass correlation coefficient (ICC) for average e′ was 0.976 (95% CI: 0.958–0.986). Inter-observer ICC was 0.969 (95% CI: 0.946–0.982). These results indicate excellent intra- and inter-observer reproducibility for the average e′ measurement. We measured both septal and lateral mitral annular e′ velocities and calculated the average e′, and adopted age-specific thresholds to define reduced average e′: < 9 cm/s for participants aged <40 years, < 7 cm/s for 40–65 years, and < 6.5 cm/s for those over 65 years; left atrial volume index (LAVI) was measured for all subjects, with LAVI > 34 mL/m^2^ defined as abnormal. Notably, tricuspid regurgitation (TR) velocity is not incorporated into the diagnostic criteria of the 2025 ASE update. In addition, only mild tricuspid regurgitation was observed in this community-based cohort and no participant had TR velocity reaching the threshold of ≥ 2.8 m/s, so this parameter was not included in our diagnostic algorithm. We applied a pre-specified rule to classify DD when parameters were discordant: participants with reduced average e′ and at least one abnormal auxiliary marker, or those with preserved average e′ and at least two abnormal auxiliary markers were diagnosed with DD, and the auxiliary markers included abnormal mitral E/a ratio (≤ 0.8 or ≥ 2), E/e′ > 14, and LAVI > 34 mL/m². Subjects who did not meet the above criteria were classified as having normal diastolic function (5, 20).

### Covariate assessment

To control for potential confounding, the following variables were adjusted for in our models:

Demographics: Age, sex, and race/ethnicity.

Lifestyle Factors and biochemical profiles: Smoking status, alcohol consumption, total cholesterol (TC), TG, low-density lipoprotein cholesterol (LDL-C), high-density lipoprotein cholesterol (HDL-C).

Clinical Profiles: systolic blood pressure (SBP), and medical history (including hypertension, stroke, diabetes, coronary heart disease). All staff responsible for blood pressure measurement received unified standardized training covering standard resting requirements, cuff placement, operating specifications of the Omron HBP-1120U automatic oscillometric sphygmomanometer and standardized reading recording rules before initiating subject enrollment. Inter-rater reliability was validated in advance by parallel blood pressure testing on 30 volunteer participants by all operators; the intraclass correlation coefficient for SBP measurements exceeded 0.90, indicating excellent inter-rater measurement consistency.

### Statistical analysis

Continuous data are reported as mean ± standard deviation (SD) or median (interquartile range, IQR), while categorical data are presented as frequencies (percentages). The association between TyG-WC and DD was estimated using three sequential multivariable logistic regression models: Model 1: crude (unadjusted). Model 2: Adjusted for demographic variables (age, sex). Model 3: Fully adjusted for Model 2 variables plus TC, TG, LDL-C, HDL-C, SBP, smoking, alcohol use, and history of stroke, history of hypertension, history of diabetes, and history of coronary heart disease.

Restricted cubic splines (RCS) with three knots (10th, 50th, and 90th percentiles) were employed to detect potential non-linear associations. Robust regression was used to evaluate the linear relationship between TyG-WC and continuous echocardiographic parameters (e’ and E/e’). Additionally, we performed subgroup and interaction analyses stratified by age, sex, current smoking, and stroke history etc. For predictive modeling, the Least Absolute Shrinkage and Selection Operator (LASSO) was used for variable selection. Selected features were then integrated into a random forest model, with performance assessed via the area under the curve (AUC). Shapley additive explanations (SHAP) were used to quantify the relative contribution of each predictor. Statistical analyses were performed using R (version 4.5.1) and SAS (version 9.4). A two-sided *P* < 0.05 was considered the threshold for statistical significance.

## Results

### Baseline characteristics

**Table 1** outlines the clinical and demographic profiles of 1,413 subjects, stratified by a TyG-WC threshold of 776.5. Participants in the high TyG-WC category displayed a more severe metabolic profile, marked by significantly elevated TG, glucose (both *P* < 0.001), and TC levels (*P* = 0.002) compared to those in the low group. HDL-C was notably lower in the high TyG-WC group (*P* < 0.001), while LDL-C levels showed no statistical difference between cohorts (*P* = 0.140). Furthermore, the high TyG-WC group had significantly higher BMI and waist circumference (*P* < 0.001). In terms of echocardiographic data, the high TyG-WC group was associated with a higher Mitral Valve E/e’ ratio (*P* = 0.020) and substantially lower velocities for lateral and septal e’ (*P* < 0.001). Comorbidity analysis revealed a higher prevalence of hypertension (47% vs. 32%) and diabetes (13% vs. 4.7%) in the high TyG-WC group (both *P* < 0.001). However, the frequency of stroke and coronary heart disease did not vary significantly (*P* = 0.917 and 0.645). The overall incidence of Diastolic Dysfunction was significantly greater in the high TyG-WC group compared to the low group (39% vs. 27%, *P* < 0.001).

**Table 1:** Baseline characteristics by TyG-WC group (cutoff=776.5).

Variable	Overall (N = 1413)^1^	Low TyG-WC N = 638^1^	High TyG-WC N = 775^1^	P-value^2^
Age (years)	60.00 (53.00, 67.00)	61.00 (54.00, 68.00)	59.00 (53.00, 67.00)	0.067
Sex (Male)	561 (40%)	213 (33%)	348 (45%)	<0.001
Current smoking	298 (21%)	119 (19%)	179 (23%)	0.049
Alcohol use	128 (9.1%)	29 (4.5%)	99 (13%)	<0.001
Systolic blood pressure (mmHg)	151.50 (137.50, 162.50)	149.50 (133.50, 162.50)	152.00 (142.00, 163.00)	<0.001
Diastolic blood pressure (mmHg)	90.50 (81.00, 100.50)	87.50 (79.00, 99.50)	92.50 (83.00, 101.50)	<0.001
Waist circumference (cm)	88.00 (81.00, 95.00)	80.50 (76.00, 85.00)	94.00 (90.00, 99.00)	<0.001
Body mass index (kg/m²)	25.73 (23.58, 28.10)	23.65 (21.95, 25.12)	27.64 (25.88, 29.51)	<0.001
Total cholesterol (mmol/L)	5.26 (4.28, 6.46)	5.06 (4.14, 6.44)	5.40 (4.43, 6.49)	0.002
Low-density lipoprotein cholesterol (mmol/L)	2.97 (2.19, 4.16)	2.92 (2.11, 4.16)	3.01 (2.28, 4.16)	0.140
High-density lipoprotein cholesterol (mmol/L)	1.40 (1.21, 1.62)	1.47 (1.30, 1.74)	1.33 (1.16, 1.53)	<0.001
Glucose (mmol/L)	5.60 (5.30, 6.30)	5.40 (5.20, 5.90)	5.90 (5.40, 6.80)	<0.001
Triglycerides (mmol/L)	1.65 (1.17, 2.31)	1.28 (0.97, 1.77)	1.98 (1.51, 2.74)	<0.001
Left ventricular mass index (g/m²)	77.28 (66.45, 89.21)	77.11 (66.00, 88.94)	77.96 (66.67, 89.49)	0.393
Left ventricular ejection fraction (%)	68.00 (63.00, 72.00)	68.00 (64.00, 72.00)	68.00 (62.00, 71.00)	0.008
Left atrial volume index (ml/m²)	27.36 (21.39, 33.85)	27.44 (21.30, 33.99)	27.35 (21.56, 33.81)	0.718
Relative wall thickness	0.37 (0.33, 0.41)	0.37 (0.33, 0.40)	0.37 (0.34, 0.41)	0.162
Mitral Valve E/e’ ratio	8.13 (6.33, 10.21)	7.92 (6.23, 9.98)	8.40 (6.50, 10.39)	0.020
Mitral Valve lateral e’ (cm/s)	9.45 (7.50, 11.56)	9.80 (7.96, 12.01)	9.10 (7.28, 11.13)	<0.001
Mitral Valve septal e’ (cm/s)	6.93 (5.62, 8.80)	7.32 (5.83, 9.10)	6.66 (5.41, 8.61)	<0.001
Hypertension	562 (40%)	201 (32%)	361 (47%)	<0.001
Diabetes mellitus	127 (9.0%)	30 (4.7%)	97 (13%)	<0.001
Stroke	157 (11%)	72 (11%)	85 (11%)	0.917
Coronary heart disease	83 (5.9%)	40 (6.3%)	43 (5.5%)	0.645
Diastolic dysfunction	477 (34%)	175 (27%)	302 (39%)	<0.001

^1^ Median (Q1, Q3), n (%); ^2^Wilcoxon rank sum test; Pearson’s Chi-squared test; TyG-WC, Triglyceride-glucose waist circumference.

### Logistic regression of TyG-WC with diastolic dysfunction

The association between TyG-WC and DD is detailed in **Table 2**. After adjusting for potential confounders in the full model (Model 3), each 1-unit increase in TyG-WC was independently associated with an OR of 1.002 (95% CI: 1.001-1.004, *P* < 0.001). When analyzed as a binary variable, participants in the high TyG-WC group faced a significantly increased risk of DD (OR = 1.656, 95% CI: 1.266-2.166, *P* < 0.001) relative to the low group.

**Table 2:** Logistic regression of TyG-WC with DD.

Exposure	Model 1 (N = 1413)		Model 2 (N = 1413)		Model 3 (N = 1413)	
	OR (95% CI)	P-value	OR (95% CI)	P-value	OR (95% CI)	P-value
TyG-WC (per 1-unit increase)	1.002 (1.001, 1.003)	<0.001	1.003 (1.002, 1.004)	<0.001	1.002 (1.001, 1.004)	<0.001
TyG-WC group (High vs Low; Cutoff=776.5)	1.689 (1.347, 2.118)	<0.001	1.854 (1.466, 2.344)	<0.001	1.656 (1.266, 2.166)	<0.001

Model 1: unadjusted. Model 2: adjusted for age, sex. Model 3: adjusted for all covariates (age, sex, current smoking, alcohol use, SBP, TC, LDL-C, HDL-C, TG, hypertension, diabetes, CHD, stroke); TyG-WC, Triglyceride-glucose waist circumference; OR, odds ratio; CI, confidence interval.

### Evaluation of predictive value

**Figure 1:**
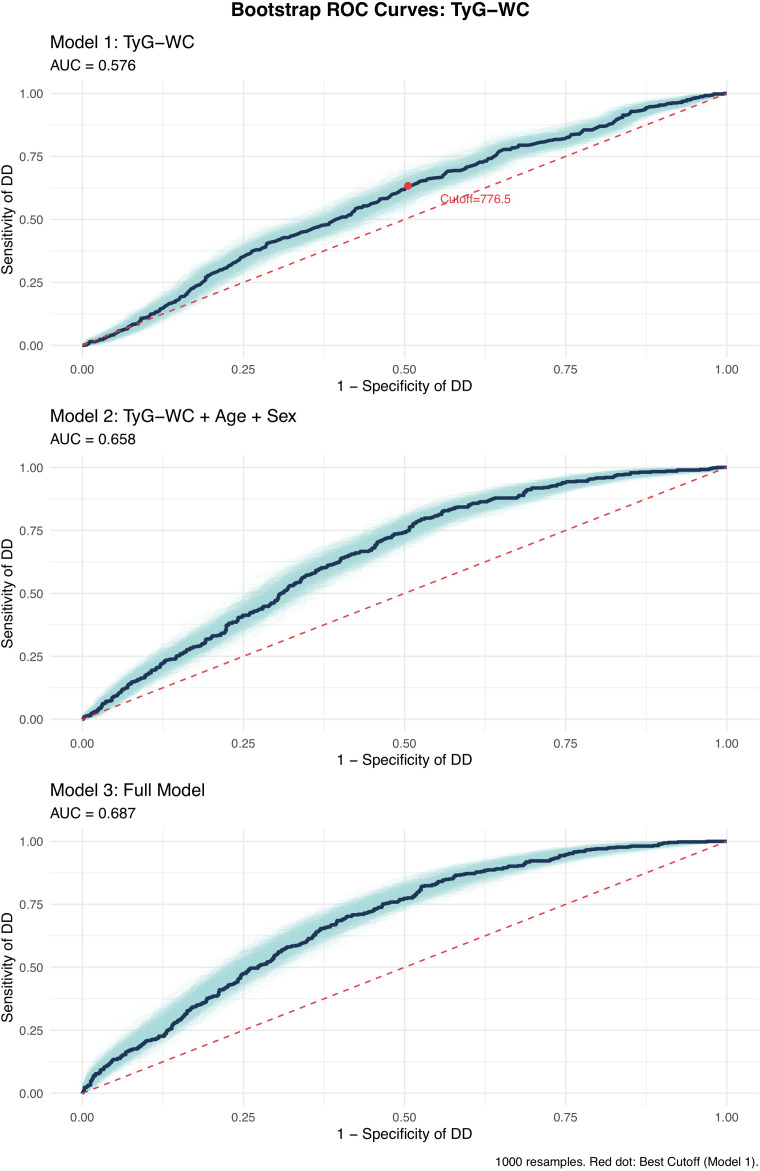
Receiver operating characteristic (ROC) curves for TyG-WC predicting diastolic dysfunction. ROC curves with 1,000 bootstrap resamples illustrating the discriminative performance of TyG-WC for diastolic dysfunction (DD) across three nested logistic regression models. Model 1 (TyG-WC only); Model 2 (TyG-WC + age + sex); Model 3 (TyG-WC + age + sex + SBP + TC + LDL-C + HDL-C + TG + current smoking + alcohol use + hypertension + diabetes + CHD + stroke). The red dot on the Model 1 curve denotes the optimal cutoff determined by the Youden index. Light blue lines represent individual bootstrap iterations; the dark blue line represents the original ROC curve. The red dashed diagonal line indicates the reference line of no discrimination (AUC = 0.5).

**Figure 1** presents the ROC curves evaluating the predictive capacity of TyG-WC-based models. Model 1, which included only TyG-WC, showed an AUC of 0.576. The addition of demographic variables (Model 2: age and sex) improved the AUC to 0.658. The comprehensive full model (Model 3) demonstrated the best performance, achieving an AUC of 0.687.

### Dose-response relationship between TyG-WC and diastolic dysfunction

**Figure 2:**
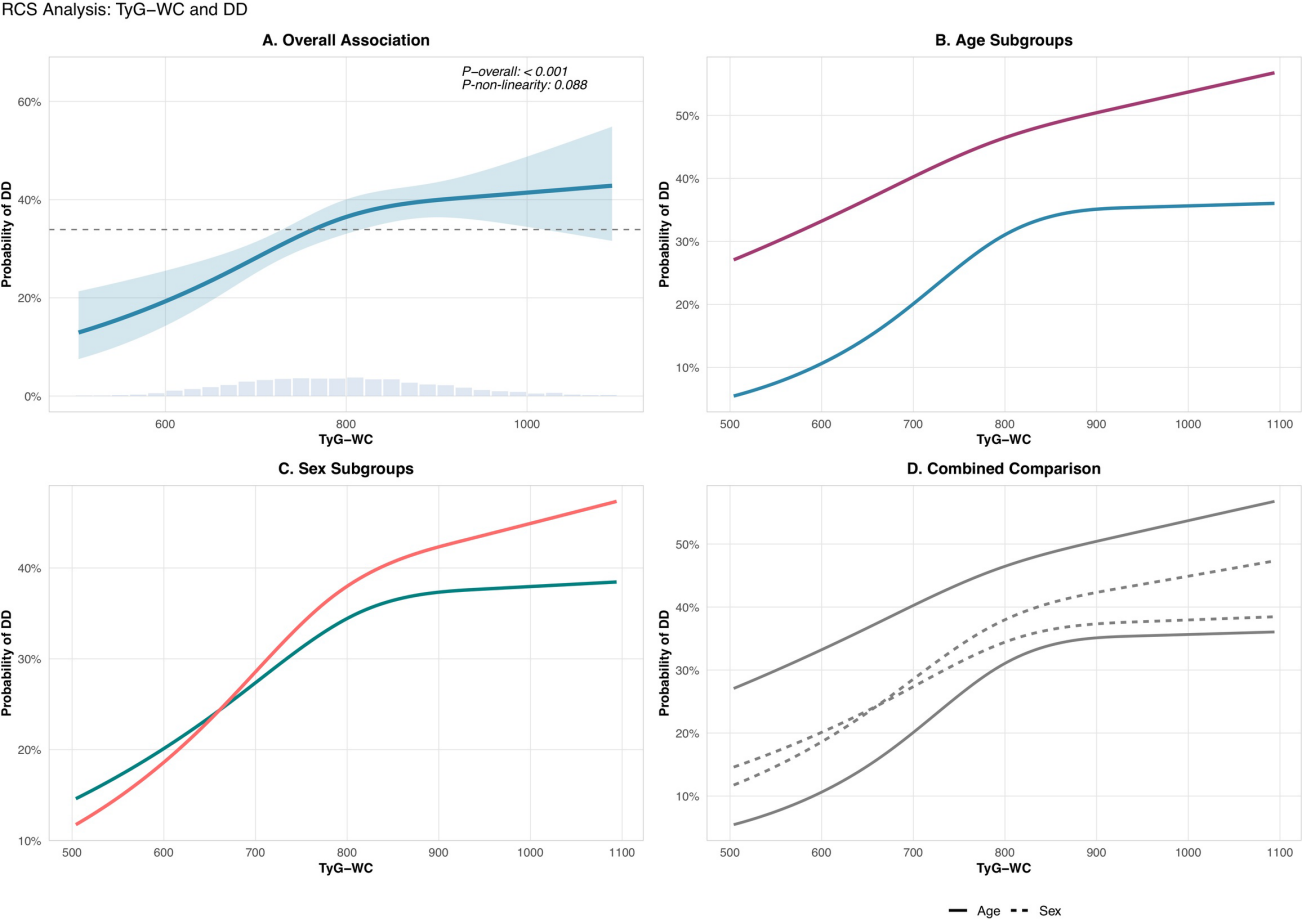
Restricted cubic spline (RCS) analysis of the association between TyG-WC and diastolic dysfunction. **(A)** Overall dose-response relationship between TyG-WC and the predicted probability of DD, adjusted for age, sex, SBP, TC, LDL-C, HDL-C, TG, current smoking, alcohol use, hypertension, diabetes, CHD, and stroke. The solid blue line represents the fitted RCS curve with 3 knots placed at the 10th, 50th, and 90th percentiles of TyG-WC; the shaded area represents the 95% confidence interval. The gray histogram depicts the distribution of TyG-WC. The dashed horizontal line indicates the overall prevalence of DD. P-overall (< 0.001) and P for non-linearity (0.088) are annotated. **(B)** Age-stratified RCS curves: < 65 years (blue) vs. ≥ 65 years (orange). **(C)** Sex-stratified RCS curves: male (teal) vs. female (coral). **(D)** Combined comparison of age and sex subgroup RCS curves.

The RCS curve (**Figure 2A**) demonstrated a significant overall association between TyG-WC and the outcome (*P*-overall:<0.001). The P for non-linearity was 0.08, indicating no statistically significant departure from linearity; the results therefore support an approximately linear dose-response relationship. The probability of DD rose progressively with increasing TyG-WC levels across the observed range. This positive correlation remained consistent across various age and sex strata (**Figures 2B–D**).

### Correlation between TyG-WC and echocardiographic parameters

**Figure 3:**
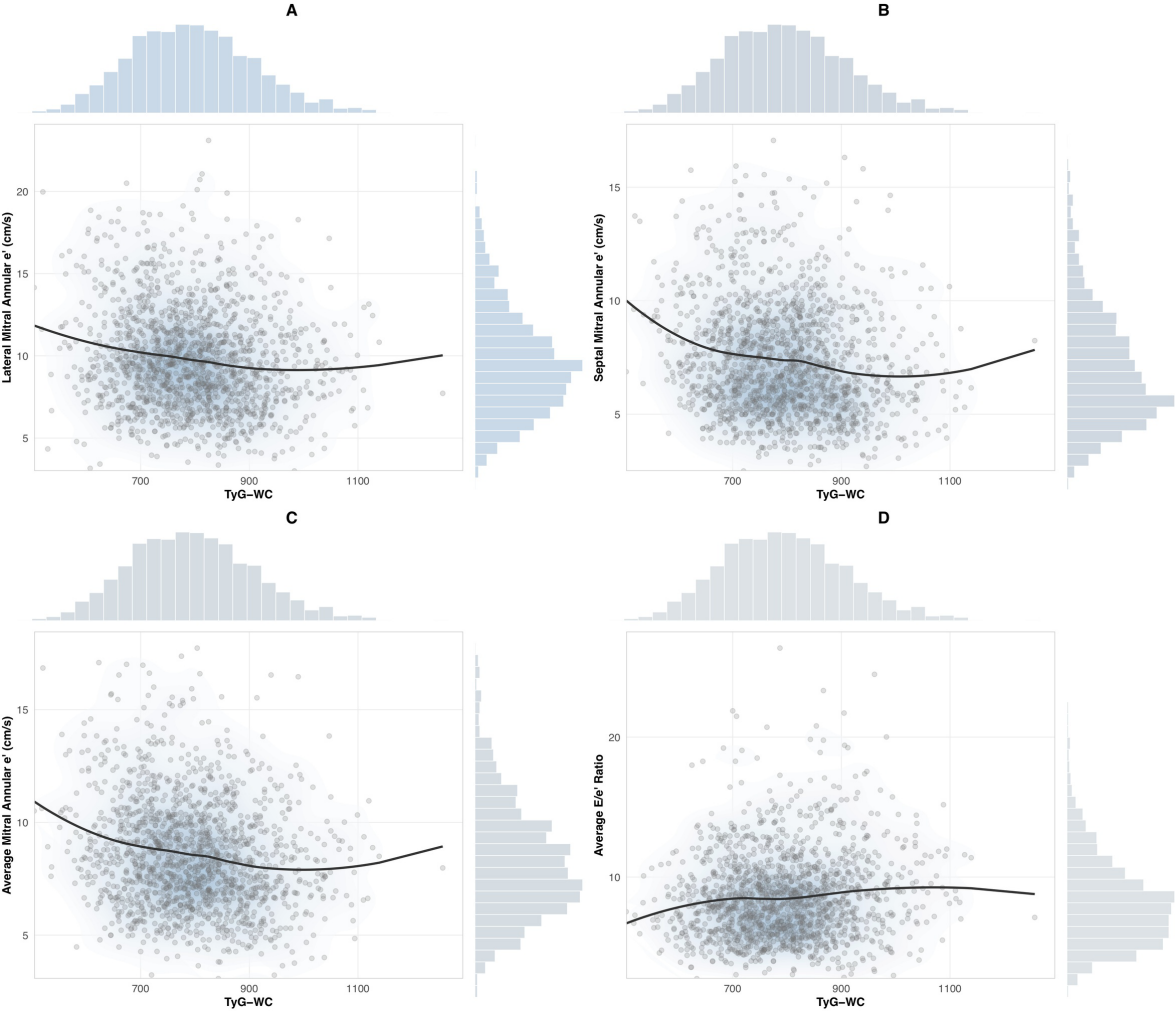
Scatter-density plots of the association between TyG-WC and echocardiographic parameters of diastolic function. Two-dimensional density scatter plots with LOESS smoothing (black line) showing the relationship between TyG-WC and four echocardiographic indicators: **(A)** lateral mitral annular e′ velocity, **(B)** septal mitral annular e′ velocity, **(C)** average mitral annular e′ velocity, and **(D)** mitral E/e′ ratio. Color gradients represent local point density from low (dark blue) to high (yellow). Marginal histograms along the top and right axes display the univariate distribution of each variable. All analyses are based on complete cases (N = 1,413).

Scatter plots in **Figure 3** illustrate the relationship between TyG-WC and parameters of diastolic function. Rising TyG-WC levels were linked to an initial decline in lateral, septal, and average e’ velocities, followed by a slight stabilization or recovery at higher indices (**Figures 3A–C**). Concurrently, the average E/e’ ratio exhibited a general fluctuating upward trend as TyG-WC increased (**Figure 3D**).

### Subgroup analysis and interaction assessment

**Figure 4:**
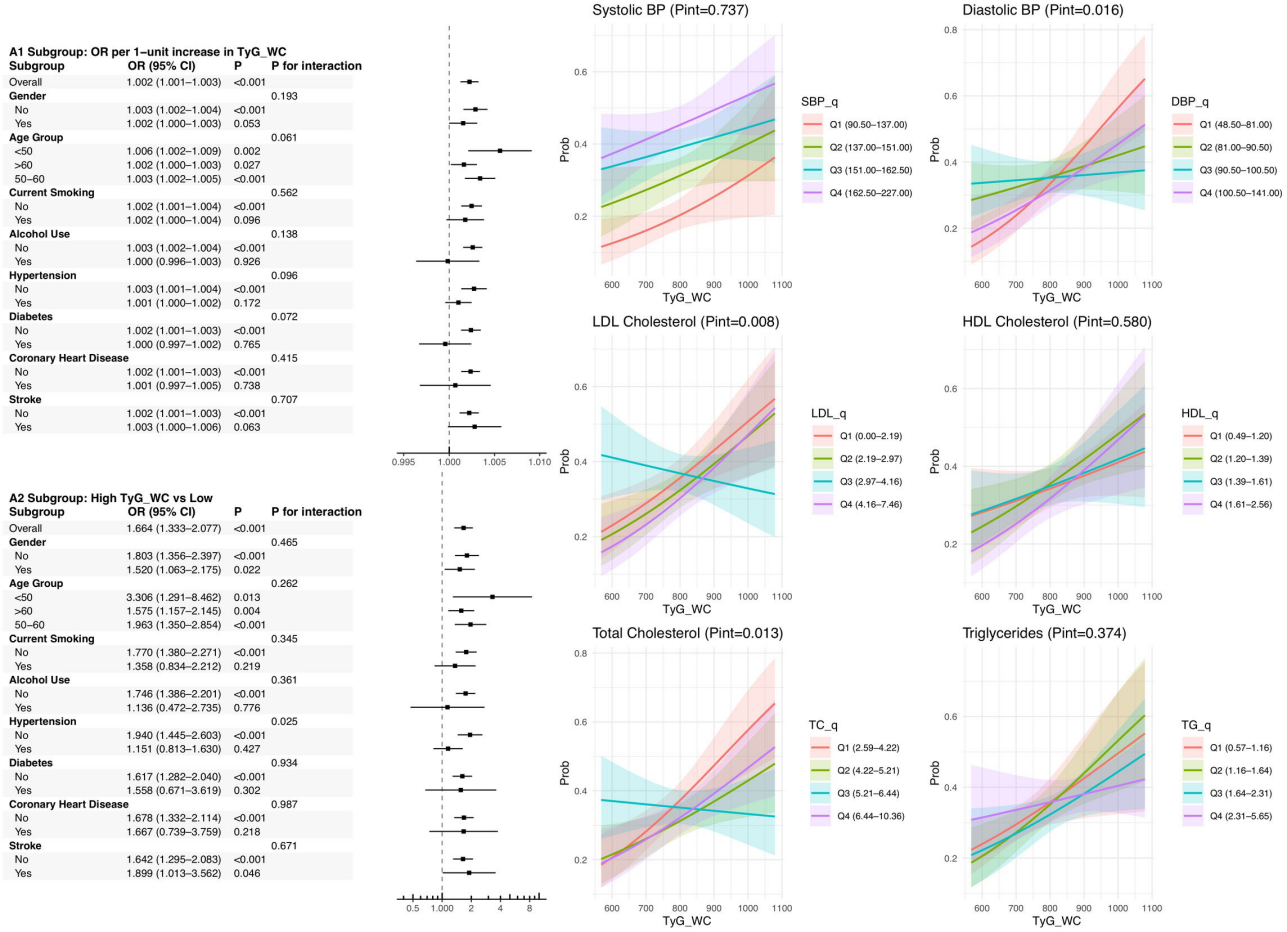
Subgroup analysis and interaction assessment for the association between TyG-WC and diastolic dysfunction. Forest plots displaying adjusted odds ratios (OR) and 95% confidence intervals for the association between TyG-WC and DD across prespecified subgroups. Panel A1: OR per 1-unit increase in continuous TyG-WC. Panel A2: OR for high vs. low TyG-WC. Subgroups include sex (male/female), age (< 65/≥ 65 years), current smoking (no/yes), alcohol use (no/yes), hypertension (no/yes), diabetes (no/yes), coronary heart disease (no/yes), and stroke (no/yes). P for interaction was derived from likelihood ratio tests comparing models with and without the interaction term. The dashed vertical line at OR = 1.0 indicates the null effect. Lower panels display predicted probability curves illustrating interaction effects between TyG-WC and key continuous covariates (SBP, DBP, LDL-C, HDL-C, TC, TG) across quartiles.

Subgroup analysis (**Figure 4**) revealed that continuous TyG-WC was significantly linked to the outcome in some participants. When analyzed as a continuous variable, higher TyG_WC was uniformly associated with increased risk across nearly all strata (all *P* < 0.05), with no significant interactions detected (all *P* for interaction > 0.05). When evaluated categorically (High vs. Low), a significant interaction was observed with hypertension status (*P*_interaction = 0.025); the risk was more pronounced in non-hypertensive individuals (OR = 1.940, 95% CI: 1.445–2.603) than in those with hypertension (OR = 1.151, 95% CI: 0.813–1.630).

### Machine learning model analysis

**Figure 5:**
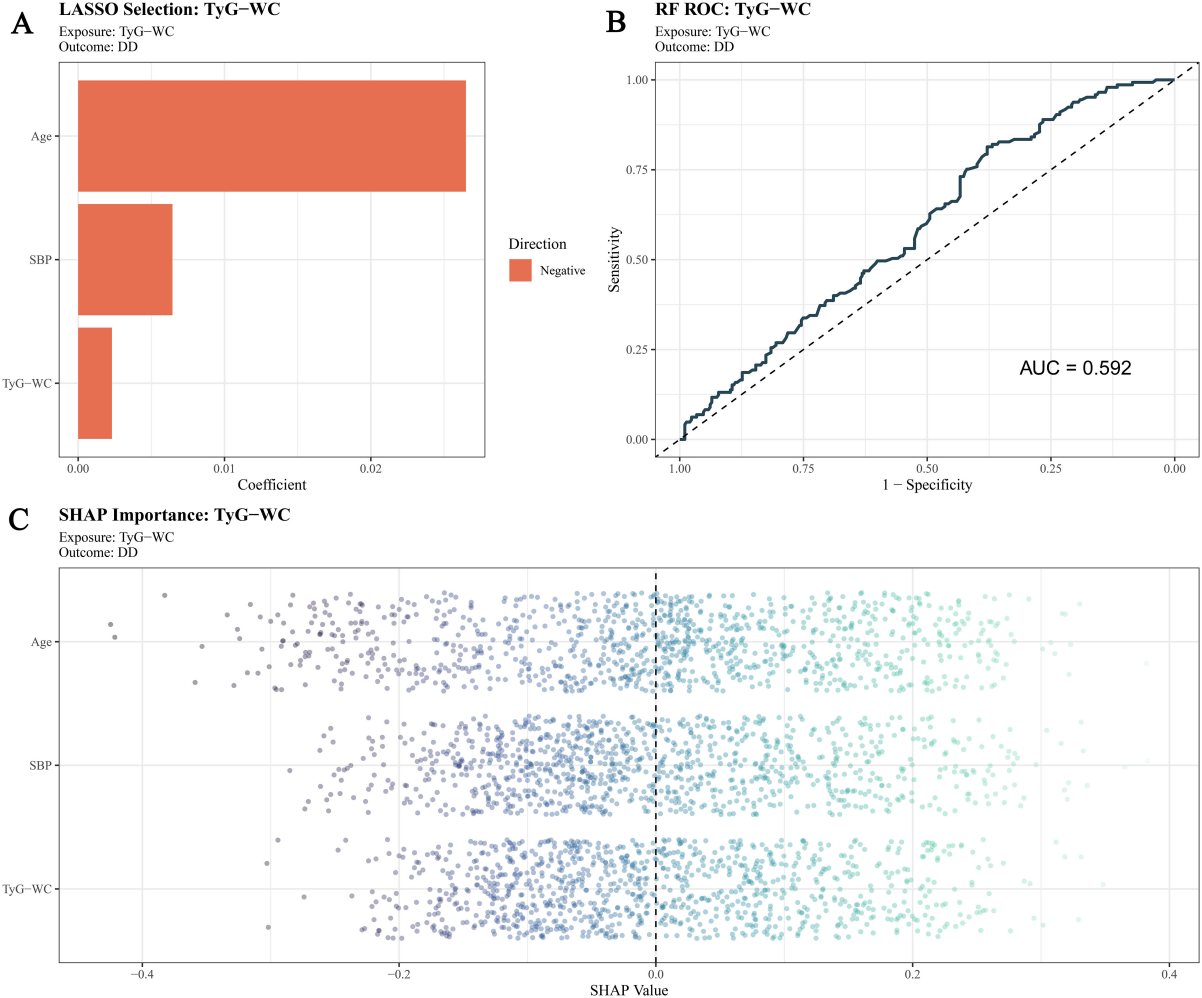
Machine learning analysis: LASSO feature selection, random forest prediction, and SHAP interpretation for TyG-WC and diastolic dysfunction. **(A)** LASSO regression coefficient profile. Variables with non-zero coefficients were selected by the optimal lambda (lambda.1se) in a least absolute shrinkage and selection operator (LASSO) model with TyG-WC forced into the model (penalty factor = 0). Bar color indicates the direction of association (blue/red = negative/positive coefficient). **(B)** Receiver operating characteristic curve for the random forest model constructed using LASSO-selected features, evaluated on the held-out test set. AUC is annotated on the plot. **(C)** SHAP (SHapley Additive exPlanations) bee swarm plot showing the distribution of SHAP values for each feature in the random forest model. Features are ranked by mean absolute SHAP value (descending). Each point represents one observation; color encodes the SHAP value (blue = low, red = high), with higher SHAP values indicating a greater contribution to the predicted probability of DD.

Machine learning results are displayed in **Figure 5**. The LASSO algorithm identified TyG-WC, SBP, and age as the most influential features, with all three showing negative coefficients in the predictive selection. The Random Forest model achieved an AUC of 0.592 for identifying DD. SHAP values ranked feature importance as age > SBP > TyG-WC.

## Discussion

This community-based study involving 1,413 middle-aged and elderly individuals demonstrates a robust and independent association between the TyG-WC index and DD, as adjudicated by the latest 2025 ASE age-stratified criteria (1). The present study identifies the TyG-WC index as a robust and independent predictor of DD in middle-aged and elderly individuals, even after accounting for established cardiovascular risk factors. Our analysis yielded several pivotal observations: 1) RCS model revealed a significant linear progression in DD probability corresponding to rising TyG-WC levels (*P* < 0.001), with 776.5 identified as the optimal clinical threshold. While the index achieved a standalone discriminative capacity (AUC = 0.576), its integration into the comprehensive model enhanced predictive accuracy to an AUC of 0.687. 2) multivariable adjustment confirmed that each incremental unit of TyG-WC correlates with a heightened likelihood of DD (OR = 1.002, 95% CI: 1.001–1.004, *P* < 0.001), while those exceeding the 776.5 cutoff exhibited a 65.6% elevation in risk (*P* < 0.001) relative to the low-TyG-WC group. 3) higher TyG-WC were significantly associated with adverse echocardiographic shifts, specifically diminished septal e’ velocities and increased E/e’ ratios. These results underscore that TyG-WC, by capturing the synergistic pathological burden of insulin resistance and visceral obesity, serves as a sensitive surrogate for detecting subclinical myocardial relaxation impairment and elevated filling pressures. Consequently, TyG-WC emerges as a valuable cardiometabolic indicator for early-stage DD identification and risk stratification.

The robust association between the TyG-WC index and DD observed in this study aligns with and extends the growing body of evidence highlighting the limitations of traditional adiposity and metabolic markers. While conventional metrics like BMI primarily reflect general adiposity, they often fail to account for the deleterious effects of regional fat distribution and systemic metabolic dysregulation (11, 21). Our findings are consistent with previous investigations suggesting that visceral adiposity—captured here by waist circumference—is a more potent driver of adverse cardiac remodeling and subclinical heart failure than total body mass (22, 23). Furthermore, the observed performance of TyG-WC is in line with earlier reports demonstrating that the TyG index serves as a reliable surrogate for insulin resistance, which is intrinsically linked to myocardial stiffness (7, 24). By integrating these two pathological dimensions, the TyG-WC index appears to offer superior risk identification for DD compared to either TyG or waist circumference alone, a trend reflected in our ROC analysis and machine learning variable importance rankings. This supports the concept that a “metabolic-mechanical” synergy—combining insulin-related biochemical stress with the pro-inflammatory burden of abdominal obesity—provides a more nuanced characterization of the systemic environment conducive to early cardiac functional decline (23, 24). Moreover, our results, which demonstrate a linear dose-response relationship through restricted cubic spline analysis and stability across hypertensive and diabetic subgroups, bolster the evidence for using integrated indices to detect subclinical myocardial impairment (25, 26).

The stronger association observed among individuals without hypertension may reflect several mechanisms. First, a ceiling effect might exist, where the incremental risk from TyG-WC is diminished once structural and hemodynamic damage from established hypertension is already present. Second, medication confounding could attenuate the association, as antihypertensive therapies and associated metabolic management modify clinical parameters. Third, TyG-WC may more accurately capture early metabolic vulnerability before the development of overt vascular disease, whereas multiple competing pathways in hypertensive patients could obscure its specific impact. Consequently, these subgroup findings remain exploratory and hypothesis-generating.

Several potential biological mechanisms may explain the link between elevated TyG-WC and DD. Insulin resistance—a core element of the TyG component—drives systemic low-grade inflammation, oxidative stress, and coronary microvascular endothelial dysfunction, which reduces nitric oxide bioavailability (24, 25). Decreased nitric oxide signaling leads to hypophosphorylation of titin and enhanced myocardial collagen cross-linking, both hallmarks of increased ventricular stiffness and delayed relaxation (27, 28). The synergy between insulin resistance and visceral adiposity may also accelerate lipotoxicity, impairing cardiomyocyte calcium handling and mitochondrial function (29). In parallel, insulin resistance and central obesity promote arterial stiffening through vascular smooth muscle cell proliferation, advanced glycation end-product (AGE)-mediated cross-linking of elastin and collagen, and endothelial nitric oxide synthase (eNOS) uncoupling, while perivascular adipose tissue dysfunction—characterized by pro-inflammatory adipokine secretion and reduced adiponectin—further reduces arterial compliance. Increased aortic stiffness elevates pulse wave velocity, causing earlier return of reflected pressure waves that augment central systolic load, impair LV relaxation, and raise LV filling pressures—key hemodynamic features of DD (30–32). Together, these interconnected myocardial and vascular mechanisms provide a biologically plausible framework for the observed TyG-WC–DD association.

From a clinical perspective, TyG-WC holds significant promise as a pragmatic screening tool. It is derived from routine clinical measurements—fasting triglycerides, glucose, and WC—making it cost-effective and easily accessible in primary care settings (33). Given the increasing prevalence of subclinical heart failure in aging populations, TyG-WC could help clinicians identify high-risk individuals who require earlier echocardiographic evaluation or more aggressive metabolic intervention. Utilizing age-stratified thresholds in accordance with the 2025 ASE guidelines ensures that such screening remains accurate across different age groups, potentially forestalling the progression from subclinical DD to symptomatic heart failure (5, 20, 33).

The strengths of this study include the use of a large-scale community cohort and a systematic analytical approach that combined multivariable regression, non-linear modeling, and machine learning techniques. However, several limitations must be considered. First, the cross-sectional design prevents us from establishing a definitive causal relationship between TyG-WC and the development of DD. Second, while we adjusted for a wide range of confounders, the possibility of residual confounding from unmeasured variables-including dietary patterns, physical activity, and medications with cardiometabolic effects (e.g., SGLT2 inhibitors, GLP-1 receptor agonists, and statins)-cannot be excluded. Third, this study was conducted in a specific geographic population, which may limit the generalizability of our findings to other ethnic groups. Finally, TyG-WC is an indirect surrogate for insulin resistance and has not been validated against gold-standard measures such as the hyperinsulinemic-euglycemic clamp or HOMA-IR in this cohort.

Future research should prioritize longitudinal studies to validate the TyG-WC index’s ability to predict the progression from subclinical impairment to overt HFpEF and cardiovascular mortality. Mechanistic research involving animal models or advanced molecular imaging is needed to further elucidate the specific pathways through which this integrated metabolic stress induces myocardial fibrosis and microvascular rarefaction. Furthermore, randomized controlled trials are warranted to determine whether interventions targeting insulin resistance and central adiposity can effectively reverse diastolic dysfunction, enabling personalized strategies for heart failure prevention.

In conclusion, an elevated TyG-WC index is independently associated with an increased prevalence of DD and impaired myocardial relaxation in middle-aged and older adults. These results suggest that TyG-WC could serve as a simple yet effective screening tool for the early detection of subclinical heart failure risk. Future longitudinal studies are warranted to determine whether targeting reductions in TyG-WC through metabolic management can effectively prevent the progression from diastolic impairment to symptomatic heart failure.

## Data Availability

The raw data supporting the conclusions of this article will be made available by the authors, without undue reservation.
